# Aryl hydrocarbon receptor modulates stroke-induced astrogliosis and neurogenesis in the adult mouse brain

**DOI:** 10.1186/s12974-019-1572-7

**Published:** 2019-10-12

**Authors:** Wan-Ci Chen, Li-Hsin Chang, Shiang-Suo Huang, Yu-Jie Huang, Chun-Lien Chih, Hung-Chih Kuo, Yi-Hsuan Lee, I-Hui Lee

**Affiliations:** 1 Department and Institute of Physiology, National Yang-Ming University, No.155, Sec. 2, Linong Street, Beitou District, Taipei, 11217 Taiwan; 2 Institute of Brain Science, National Yang-Ming University, Taipei, Taiwan; 3 Brain Research Center, National Yang-Ming University, Taipei, Taiwan; 4 Department of Pharmacology, Institute of Medicine, Chung-Shan Medical University, Taichung, Taiwan; 5 Cheng Hsin General Hospital, Taipei, Taiwan; 6 Stem Cell Program, Institute of Cellular and Organismic Biology, Academia Sinica, Taipei, Taiwan; 7 Division of Cerebrovascular Diseases, Neurological Institute, Taipei Veterans General Hospital, No.201, Sec. 2, Shipai Rd., Beitou District, Taipei, 11217 Taiwan

**Keywords:** Stroke, Aryl hydrocarbon receptor, Gliosis, Neurogenesis, Inflammation, Neural stem cells

## Abstract

**Background:**

The aryl hydrocarbon receptor (AHR) is a ligand-dependent transcription factor activated by environmental agonists and dietary tryptophan metabolites for the immune response and cell cycle regulation. Emerging evidence suggests that AHR activation after acute stroke may play a role in brain ischemic injury. However, whether AHR activation alters poststroke astrogliosis and neurogenesis remains unknown.

**Methods:**

We adopted conditional knockout of AHR from nestin-expressing neural stem/progenitor cells (AHRcKO) and wild-type (WT) mice in the permanent middle cerebral artery occlusion (MCAO) model. WT mice were treated with either vehicle or the AHR antagonist 6,2′,4′-trimethoxyflavone (TMF, 5 mg/kg/day) intraperitoneally. The animals were examined at 2 and 7 days after MCAO.

**Results:**

The AHR signaling pathway was significantly upregulated after stroke. Both TMF-treated WT and AHRcKO mice showed significantly decreased infarct volume, improved sensorimotor, and nonspatial working memory functions compared with their respective controls. AHR immunoreactivities were increased predominantly in activated microglia and astrocytes after MCAO compared with the normal WT controls. The TMF-treated WT and AHRcKO mice demonstrated significant amelioration of astrogliosis and microgliosis. Interestingly, these mice also showed augmentation of neural progenitor cell proliferation at the ipsilesional neurogenic subventricular zone (SVZ) and the hippocampal subgranular zone. At the peri-infarct cortex, the ipsilesional SVZ/striatum, and the hippocampus, both the TMF-treated and AHRcKO mice demonstrated downregulated IL-1β, IL-6, IFN-γ, CXCL1, and S100β, and concomitantly upregulated Neurogenin 2 and Neurogenin 1.

**Conclusion:**

Neural cell-specific AHR activation following acute ischemic stroke increased astrogliosis and suppressed neurogenesis in adult mice. AHR inhibition in acute stroke may potentially benefit functional outcomes likely through reducing proinflammatory gliosis and preserving neurogenesis.

**Electronic supplementary material:**

The online version of this article (10.1186/s12974-019-1572-7) contains supplementary material, which is available to authorized users.

## Introduction

The aryl hydrocarbon receptor (AHR), a ligand-activated transcription factor, is widely expressed in most mammalian tissues and evolutionarily conserved from invertebrates onward [1]. AHR is activated by a variety of ligands, including environmental pollutants such as polycyclic aromatic hydrocarbons or dioxins [2], as well as the dietary tryptophan metabolite kynurenine from indoleamine 2,3-dioxygenase (IDO) and tryptophan 2,3-dioxygenase (TDO), known for xenobiotic metabolism, immune responses, and cell cycle regulation [3–5]. The AHR dimerizes with the AHR nuclear translocator (ARNT) for nuclear import and DNA-binding to dioxin response elements (DRE), and induces the transcription of its target genes, such as the cytochrome P450 (CYP)1A1, CYP1A2, and CYP1B1 enzymes, which then degrade AHR ligands for detoxification [6]. In the mammalian nervous system, AHR is robustly expressed in embryonic neural stem cells of the developing brain [7] and decreased thereafter in the adult brain, predominantly in the cerebral cortex, hippocampus, cerebellum, and olfactory bulb [8]. Environmental pollutants of AHR ligands cause developmental deficits of the brain and increased neuropsychiatric diseases [9–11]. In the adult mouse brain, nestin-positive neural progenitor cells (NPCs) express AHR, and AHR overactivation by exogenous dioxins adversely impaired hippocampal NPC proliferation in association with impaired contextual fear memory, suggesting that AHR may be a potential transcriptional regulator of adult neurogenesis and cognitive function [12]. However, the physiological functions of AHR in the adult brain remains poorly defined. Recently, AHR has been suggested to mediate acute ischemic injury following middle cerebral artery occlusion (MCAO) in adult mice. The AHR protein levels and the AHR ligand kynurenine were rapidly increased at the peri-infarct regions and peaked at 24 to 48 h. AHR-null mice and poststroke pharmacological inhibition of AHR with the competitive antagonist, 6,2′,4′-trimethoxyflavone (TMF), after stroke reduced ischemic infarct in association with neuroprotective and antiapoptotic effects [13]. Moreover, an elevation of the kynurenine/tryptophan ratio was observed in the blood of stroke patients and correlated with infarct volume and mortality [14, 15]. Nevertheless, the AHR-dependent mechanisms underlying brain injury after acute stroke remains elusive.

AHR mediates immune and inflammatory responses in a ligand-specific, cell type-specific and disease-specific manner. We have previously demonstrated that microglial AHR mediated both proinflammatory and anti-inflammatory effects in lipopolysaccharide-activated primary cultures depending on availability of exogenous AHR ligands [16]. AHR activation can induce genomic DRE-dependent transcription of proinflammatory target genes, such as inducible nitric oxide synthase (iNOS) and cyclooxygenase-2 (COX-2), or nongenomic DRE-independent pathways [16, 17]. AHR-deficient mice suffer from multiple system pathologies, including abnormal hematopoiesis, hypertension, chronic inflammation of the gastrointestinal tract, and reduced reproductive capacity [18–22]. To avoid systemic comorbidities in AHR-deficient mice, Rothhammer et al. demonstrated that, in the experimental autoimmune encephalomyelitis mouse model, microglia-specific AHR deletion upregulated the expression of pro-inflammatory activities in astrocytes, suggesting that microglial AHR limits pathogenic activities of astrocytes in chronic autoimmune inflammation [23]. Here, we investigate the AHR-mediated neuroglial activities in the model of acute ischemic stroke using the nestin^+^ cell-specific knockout of AHR (with spared microglia) and pharmacological inhibition of AHR to assess how astrogliosis and neurogenesis are modulated in acute ischemic injury.

## Materials and methods

### Conditional knockout of AHR, MCAO, and experimental design

The animal experiments were approved by the Institutional Animal Care and Use Committee at the Taipei Veterans General Hospital (IACUC: 2016-299) and by the Experimental Animal Review Committee at National Yang-Ming University (IACUC no.: 1051216r) in Taiwan. All efforts were made to minimize animal numbers and suffering as per ARRIVE guidelines [24].

AHR^flx/flx^ were purchased with LoxP sites flanking exon 2 of the *AHR* gene (B6.129 (FVB)-AHR^tm3.1Bra^/J, The Jackson Laboratory). Nestin-Cre mice (B6.Cg-Tg (Nes-cre)1Kln/J, The Jackson Laboratory) were a kind gift from Dr. Chun-Ming Chen (Department of Life Sciences and Institute of Genome Sciences, National Yang-Ming University), and these mice express recombinase under the control of the nestin promoter. AHR^flx/flx^ mice were mated with Nestin-Cre mice to generate the heterozygous LoxP-flanked AHR (Nestin-Cre/AHR^flx/+^) mice. Then, the Nestin-Cre/AHR^flx/+^ mice were mated with AHR^flx/flx^ mice to generate Nestin-Cre/AHR^flx/flx^, i.e., AHR conditional knockout (AHRcKO) mice. AHRcKO (Nestin-Cre/AHR^flx/flx^) and their AHR^flx/flx^ littermates were used in the experiments. The integrity of LoxP and Cre gene expression was checked by agarose gel electrophoresis.

At 8–10 weeks old, male C57BL/6 wild-type (WT) mice (BioLASCO Taiwan Co., Ltd.) (*n* = 62), AHR^flx/flx^ (*n* = 32), and AHRcKO mice (*n* = 32) were subjected to permanent MCAO surgery following generalized anesthesia with intraperitoneal pentobarbital (50 mg/kg in normal saline). Briefly, we performed craniotomy and permanently cauterized the distal MCA along with simultaneous occlusion of the bilateral common carotid arteries with microaneurysm clips for 20 min to paralyze the dominant forelimb determined by the skilled forelimb reaching test (see below). The body temperature was maintained with a heat pad at 37 ± 0.5 °C with a feedback sensor. The WT mice were randomly allocated to two groups to receive the intraperitoneal administration of either vehicle (2% dimethyl sulfoxide, DMSO, Sigma-Aldrich) or the AHR antagonist TMF (5 mg/kg/day in 2% DMSO, Sigma-Aldrich) immediately after MCAO. After 2 days of MCAO, the mice were exsanguinated after anesthesia with intraperitoneal pentobarbital to harvest fresh brains for infarct size quantification (*n* = 6/group), proteins and RNA preparation (*n* = 6/ group for ipsilesional brains and *n* = 6/group for specified brain regions), and immunohistochemistry (*n* = 6/ group). After behavioral measurements for 7 days post-MCAO, the mice were euthanized and fresh brains were harvested for immunohistochemistry (*n* = 7 for the WT-Vehicle and WT-TMF groups; *n* = 8 for the AHR^flx/flx^ and AHRcKO groups). Normal-WT (*n* = 6 for IHC, *n* = 6 for ipsilesional brains, and *n* = 6 for specified brain regions) and normal-AHRcKO (*n* = 6 for IHC, *n* = 6 for ipsilesional brains, and *n* = 6 for specified brain regions) without sham surgery were used for comparison. In summary, there were in total 80 B6 mice, 32 AHR^flx/flx^ mice, and 50 AHRcKO mice used in this study.

### Behavioral tests

Observer-blinded measurements were obtained 2 days before, and at 48 h and 7 days after MCAO, including the adhesive removal test [25], the skilled forelimb reaching test [26], and the novel object recognition (TMF *n* = 7 vs. vehicle *n* = 7; AHR^flx/flx^ *n* = 8 vs. AHRcKO *n* = 8) [27]. For the adhesive removal test, the dominant (determined by the skilled forelimb reaching test; to be paretic after MCAO) forepaw was cleaned and attached with adhesive tape (0.3 × 0.4 cm). The timer was started once mice were placed in a glass cylinder. The time when the mice started to remove the tape and the time when the mice completely removed the tape were recorded. For the skilled forelimb-reaching test, the mice were pretrained for 2 weeks before MCAO to learn to retrieve and break pieces of uncooked capellini from a row of vertically oriented pieces of pasta (matrix) until reaching a maximum of 30 pieces in 30 min. The mouse was placed in an acrylic chamber (15 × 8.5 × 20 cm) with a slit (13 × 0.5 cm) at the center of the 15-cm-long side and the capellini matrix placed in front of the slit. The numbers of pasta pieces removed and successfully broken in 30 min were recorded. The novel object recognition test is based on the fact that animals are keen to interact more with a novel object than with a familiar object, which has been shown to be a sensitive index of nonspatial memory capabilities. The mice were habituated for 5 min in the test apparatus (30-cm-high, 30-cm-deep, and 30-cm-wide white rectangular open-field) for 1 day. In the acquisition trial, the mouse was placed in the chamber with two identical objects, one in the upper-left and the other in the upper-right quadrant, for 10 min/day in three consecutive days. In the test trial, the mice freely explored an upper-left familiar object and an upper-right novel object before and at 48 h and 7 days after MCAO surgery using different novel objects, respectively. Each session was recorded from above and analyzed for animal trajectories and the time of object contacts (sniffing-dependent measure included contacts the object with the forepaws while sniffing, SMART V3.0.03, Panlab). The discrimination index, i.e., individual time at novel object/(time at novel object + time at familiar object), was then calculated for retention test trials.

### Infarct volume quantification

After 48 h of MCAO, the mice (*n* = 6/group subjected to MCAO) were euthanized via intraperitoneal injection of a lethal dose of intraperitoneal pentobarbital (150 mg/kg), and their brains were harvested and sliced into standard 1 mm coronal slices using a brain matrix slicer (Jacobowitz Systems, Zivic-Miller Laboratories, Inc.). The brain slices were stained with 2% 2,3,5-triphenyltetrazolium chloride (TTC) for 30 min at 37 °C in the dark and fixed with 10% formalin at room temperature overnight. The total volume (in mm^3^) was calculated as the sum of infarct area on each slice with ImageJ (Java).

### Bromodeoxyuridine labeling and immunohistochemistry

To label proliferative cells in the brain, Bromodeoxyuridine (BrdU) solution (a thymidine analog that is incorporated into newly synthesized DNA of dividing cells) was freshly prepared and injected intraperitoneally (50 mg/kg/day) for seven consecutive days after stroke or in normal mice. The mice were transcardially perfused with normal saline, followed by 4% paraformaldehyde after 48 h (*n* = 6/group) and 7 days (*n* = 6/group) of MCAO. Then, the brains were dissected and fixed in 4% paraformaldehyde overnight. At 24 h postfixation, the brains were dehydrated in 30% sucrose in normal saline for days, sliced into 25 μm sections with a cryostat, and mounted onto poly-l-lysine-treated slides. For BrdU immunoreactivity, the slides were pretreated with 2 N HCL for 30 min at 37 °C, washed with phosphate-buffered saline (PBS) for 5 min, and then blocked with 2% donkey serum (Abcam) in Tris-buffered saline (TBS) containing 0.3% TritonX 100 (Sigma-Aldrich) for 2 h at room temperature. For immunofluorescent staining, the slides were incubated with the following primary antibodies: rat anti-BrdU (1:200, Abcam, ab6326), mouse anti-AHR (1:200, Abcam, ab2769), goat anti-Iba1 (Ionized calcium-binding adapter molecule 1, 1:200, Novus Biologicals, NB100-1028), rabbit anti-GFAP (glial fibrillary acidic protein, 1:200, Abcam, ab7260), rabbit anti-SOX2 (sex determining region Y-box 2, 1:200, Abcam, ab97959), and rabbit anti-DCX (Doublecortin, 1:200, Abcam, ab18723) overnight at 4 °C. Then, the slides were thoroughly washed and incubated with the following fluorescence-conjugated secondary antibodies: donkey anti-rat Alexa 488 (1:200, Jackson immune research), donkey anti-goat Alexa 488 (1:200, Jackson immune research), donkey anti-rabbit Alexa 488 (1:200, Jackson immune research), and donkey anti-mouse Cy3 (1:200, Jackson immune research) for 2 h at room temperature. Finally, the slides were washed and then cover-slipped with Vectashield Fluorescent Mounting Medium containing DAPI nuclear counter stain (Vector Laboratories). The images were taken with an Olympus FV1000 confocal microscope and quantified with ImageJ software. The AHR antibody specificity and intracellular distribution of immunoreactivity was demonstrated in Additional file 1: Figure S1. We quantified and averaged the densitometry of the fluorescence for AHR, Iba1, GFAP, and the cell numbers for SOX2-positive and BrdU/DCX-double positive cells from three brain sections per mice per specified location as a percentage of the total area quantified, or of the total DAPI-positive cell numbers quantified, respectively.

### Western blotting

The ipsilesional brain hemisphere (*n* = 6/group) and specified brain regions, including peri-infarct, SVZ/Striatum, and hippocampus (*n* = 6/group), was freshly dissected on ice at 48 h after MCAO, homogenized in T-PER Tissue Protein Extraction Reagent (Thermo Fisher), and centrifuged at 10,000×g for 20 min to obtain protein supernatants. After the protein concentration was determined using the NanoVue Plus Spectrophotometer, 50 μg of the protein was loaded onto 7.5%, 12%, or 15% gels, separated by SDS-PAGE electrophoresis, and transferred onto PVDF membranes. The membranes were blocked with 10% skim milk in phosphate-buffered saline Tween-20 (PBST) for 60 min, followed by incubation with the following primary antibodies: mouse anti-AHR (1:1000, Abcam, ab2769), mouse anti-ARNT (1:1000, Abcam, ab2771), mouse anti-CYP1B1 (1:1000, Santa Cruz, sc-374228), mouse anti-CYP1A2 (1:1000, Santa Cruz, sc-53,614), mouse anti-IDO (1:1000, Millipore, MAB5412), rabbit anti-S100β (1:1000, Abcam, ab52642), and rabbit anti-Neurogenin 2 (1:1000, Millipore, AB5682). Mouse anti-Neurogenin 1 (1:1000, Santa Cruz, sc-10,032), rabbit anti-CXCL1 (Chemokine (C-X-C motif) ligand 1, 1:1000, Proteintech, 12335-1-AP), and rabbit anti-β-actin (1:5000, Proteintech, 60008-1-lg) overnight at 4 °C. The membranes were washed with PBST, incubated with HRP-conjugated secondary antibodies (1:5000, Millipore) for 1 h at room temperature, and then washed again. The blots were detected with Clarity Western ECL Substrate. Finally, the protein band was normalized to the internal control level of β-actin and then analyzed as fold changes using ImageJ.

### Enzyme-linked immunosorbent assay

The levels of interleukin (IL)-1β, IL-6, and interferon-γ (IFN-γ) derived from the serum and the ipsilesional brain hemisphere (*n* = 6/group, 48 h after MCAO) were separated using a mouse enzyme-linked immunosorbent assay (ELISA) kit (BMS6002, BMS603-2, and BMS606INST, Invitrogen) according to the manufacturer’s instructions and detected by an ELISA reader at 450 nm. The concentrations of IL-1β, IL-6, and IFN-γ were calculated and displayed as pg/μg total protein.

### DRE luciferase activity assay

A luciferase reporter plasmid pGL2–3xDRE-luciferase construct driven by AHR-dependent DRE binding was used to assess AhR agonist activity. The plasmid was constructed by inserting a synthesized DNA fragment containing three DRE. To assay the reporter activity of the pGL2–3xDRE-luciferase construct, the C6 glioma cell line was transfected using the Lipofectamine 2000 system. The transfected cells were recovered in medium for 24 h. For the cell-based AHR activity assay, C6-DRE glioma cells were treated with 25% normal and ipsilesional brain lysates (*n* = 6/group) for 24 h. The treated cells were then lysed, and the luciferase activity was assayed using the ONE-Glo™ Luciferase Assay System. The luminescence was detected and analyzed using a luminometer. The relative luciferase activity of each sample was calculated by comparing the ratio to the control group.

### Real-time polymerase chain reaction array of neurogenesis genes

Total RNA was extracted from the aforementioned homogenates by using TRI Reagent®, and total RNA was reverse transcribed by using the Scientific Maxima First Strand cDNA Synthesis Kit (Thermo Scientific) to obtain cDNAs for subsequent quantitative PCR. The RT^2^ Profiler PCR neurogenesis array (QIAGEN) includes primer assays for 84 candidate genes and five housekeeping genes (*n* = 3/group) (Additional file 1: Figure S2C). The components were mixed with Fast SYBR® Green Master Mix, sample cDNA, and RNase-free water, and then added to each well of the RT^2^ Profiler PCR neurogenesis array. The real-time PCR reaction was performed using the ABI 7900HT Fast Real-Time PCR System. The average cycle threshold value was used to calculate mRNA expression levels and normalized to the housekeeping genes. Fold changes at least beyond 1 ± 0.5-fold were defined as differentially expressed compared with the control group.

### Statistical analysis

Data analysis was performed using SigmaPlot. A repeated measures analysis of variance (ANOVA) test was used for the within-subject factor “time” and between-subjects factor “group” to determine any differences in the behavioral measures, followed by Student’s *t* tests with the post hoc Bonferroni correction at different time points. To evaluate differences in the histological analyses, DRE luciferase activity assays, gene expression, and Western blotting among groups, a one-way ANOVA test was used, followed by Tukey’s post hoc test. Statistical significance was defined as a *p* value < 0.05.

## Results

### Both pharmacological inhibition and nestin^+^ cell-specific knockout of AHR attenuated brain infarctions and functional impairments

**Fig. 1:**
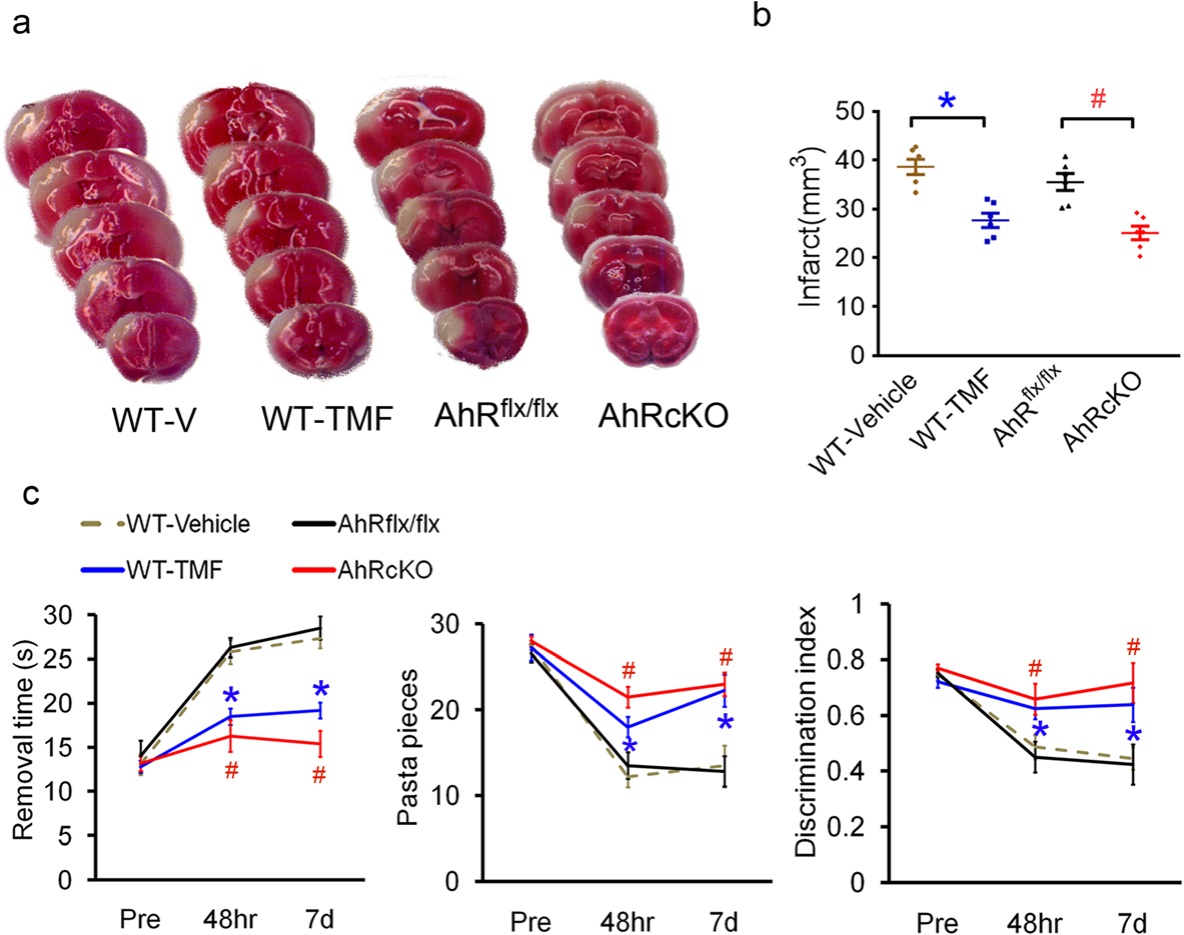
Both TMF-treated and AHRcKO mice attenuated acute cerebral infarction and functional impairments. **a** Representative brain slices with viable 2,3,5-triphenyltetrazolium chloride (TTC) staining at 48 h after permanent middle cerebral artery occlusion (MCAO) from wild-type (WT)-vehicle-treated mice, AHR antagonist TMF-treated mice, AHR^flx/flx^ mice, and AHRcKO mice. **b** The infarct volume was significantly reduced in the WT-TMF-treated group compared to the WT-vehicle-treated group, and AHRcKO mice also showed a significantly reduced infarct volume compared to AHR^flx/flx^ mice (*n* = 6/group). **c** The sensorimotor functions of the WT-TMF-treated mice compared with the WT-vehicle-treated mice (*n* = 7/group) in the adhesive removal test and the skilled forelimb-reaching pasta matrix test at 48 h and 7 days after MCAO. The novel object recognition test of nonspatial working memory was measured as the discrimination index compared with AHR^flx/flx^ mice (*n* = 8/group). The results are expressed as the means ± standard error of the mean. *p < 0.05 WT-TMF compared with WT-Vehicle. #*p* < 0.05 AHRcKO compared with AHR^flx/flx^

The aryl hydrocarbon receptor (AHR) antagonist 6,2′,4′-trimethoxyflavone (TMF)-treated wild-type (WT) and AHR conditional knockout (AHRcKO) mice exhibited a reduction in brain infarction compared with the vehicle-treated WT and AHR^flx/flx^ mice, respectively (Fig. 1a, b). Furthermore, the TMF-treated mice and AHRcKO mice took a shorter time to remove the adhesive from their affected forepaws in the adhesive removal test and successfully retrieved more pasta pieces in the skilled reaching task at 48 h and 7 days poststroke (Fig. 1c). Furthermore, in the novel object recognition test for nonspatial working memory, we found that the TMF-treated mice and AHRcKO mice explored the novel object more than the familiar object at 48 h and 7 days after middle cerebral artery occlusion (MCAO) (Fig. 1c). Thus, the AHR inhibition and nestin^+^ cell-specific knockout of AHR both attenuated acute ischemic infarction and functional deficits of the sensorimotor and hippocampus-related pattern separation performance.

### Regulation of the AHR pathway after stroke and following treatment

**Fig. 2:**
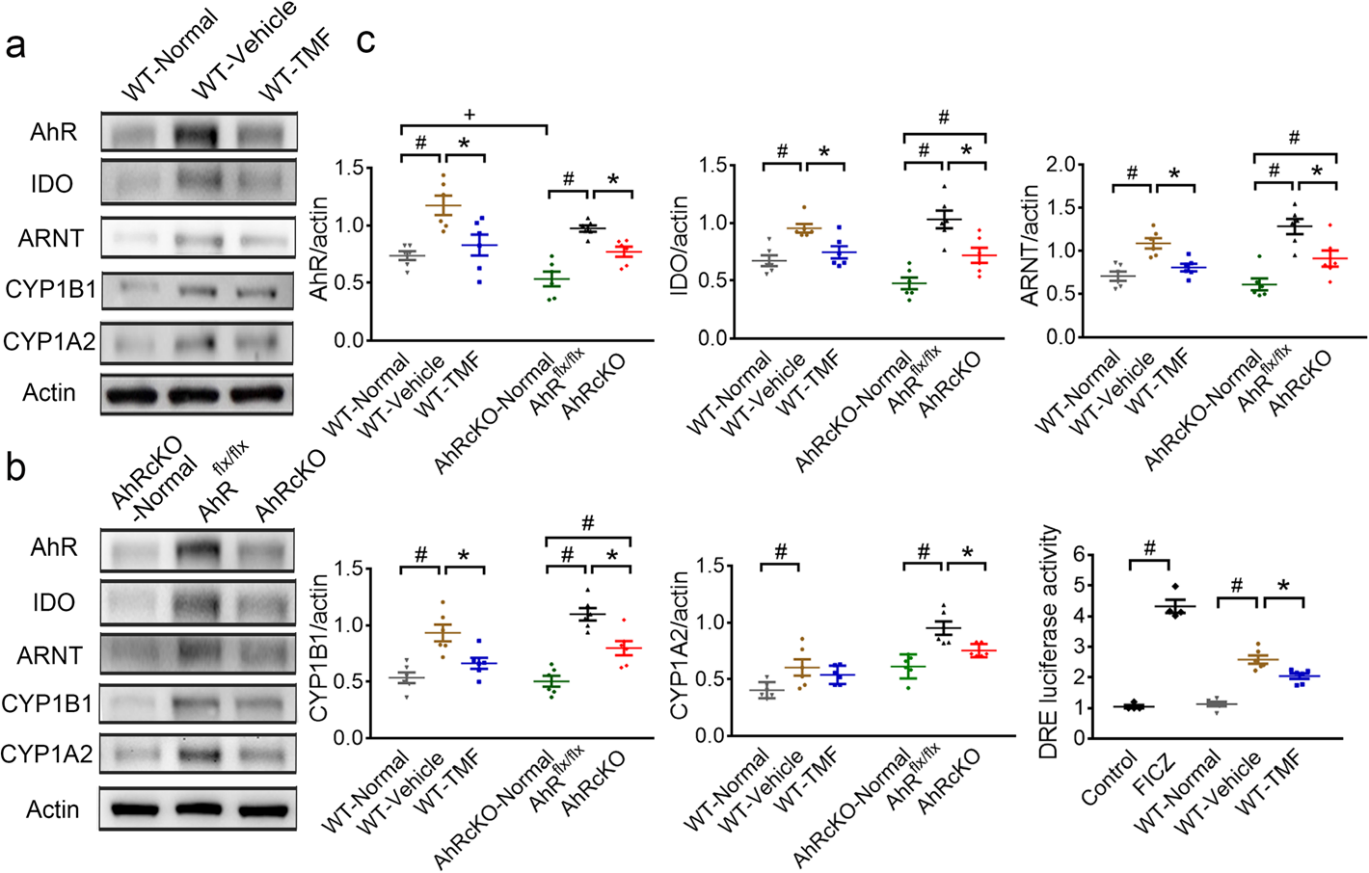
Both TMF-treated and AHRcKO mice showed attenuated AHR pathway proteins and AHR activities after MCAO. **a** Representative Western blots of the normal WT and poststroke ipsilesional hemisphere lysates from WT-vehicle and WT-TMF treated mice at 48 h after MCAO with β-actin as the loading control. **b** Representative Western blots of normal AHRcKO and ipsilesional AHR^flx/flx^ and AHRcKO brains after MCAO with β-actin as the loading control. **c** Quantitative levels of AHR pathway proteins in normal and poststroke brains from AHRcKO, WT, WT-vehicle, and WT-TMF mice (*n* = 6/each group). The AHR, IDO, ARNT, CYP1B1, and CYP1A2 proteins were increased in the WT-vehicle group after MCAO compared to the WT-normal group. The poststroke AHR^flx/flx^ group also had increased AHR, IDO, ARNT, CYP1B1, and CYP1A2 protein levels compared to the normal AHRcKO group. Compared to the WT-vehicle-treated group, the WT-TMF-treated group showed significantly decreased AHR pathway proteins after MCAO. Similarly, the AHRcKO group revealed downregulated AHR pathway proteins compared to the AHR^flx/flx^ group after MCAO. The AHR agonist activity, measured by the relative DRE-luciferase activity compared to control, was increased by treatment with the AHR agonist, FICZ, compared with control. After MCAO, the WT-vehicle-treated group showed a significant increase in AHR agonist activity compared with the normal WT group. The increased AHR agonist activity after MCAO was decreased by TMF treatment, suggesting that TMF downregulated the IDO/kynurenine/AHR activation. The results are expressed as the means ± standard error of the mean. +*p* < 0.05 AHRcKO-Normal compared with WT-Normal mice. #*p* < 0.05 compared with the respective normal controls. *p < 0.05 WT-TMF-treated mice compared with the WT-Vehicle and AHRcKO mice compared with the AHR^flx/flx^ mice

The AHR protein in the normal AHRcKO brain was significantly decreased compared with that in the normal WT mouse brain (Fig. 2). After 48 h of MCAO, the WT-Vehicle and AHR^flx/flx^ mice showed significantly increased AHR pathway proteins, including AHR, indoleamine 2,3-dioxygenase (IDO) that triggers the kynurenine pathway, AHR nuclear translocator (ARNT), cytochrome P450 (CYP)1B1, and CYP1A2. In contrast, the TMF-treated WT mice and the AHRcKO mice showed significantly downregulated AHR, IDO, ARNT, and CYP1B1 proteins at 48 h after MCAO (Fig. 2a–c). Using the glioma C6-dioxin response elements (DRE)-luciferase cell-based assay of AHR agonist activities (Fig. 2c), in which AHR activation can be quantified by DRE-induced luciferase expression, the AHR agonist 6-formylindolo(3,2-b)carbazole (FICZ) significantly increased AHR activity compared with the control. After stroke, the WT-vehicle brain lysates had significantly increased AHR activity compared with that in the normal WT brain lysates, which was reduced in the brain lysates of TMF-treated group. Taken together, AHR inhibition and the nestin^+^ cell-specific knockout of AHR downregulated AHR activation-mediated gene expression following acute ischemic stroke.

### Both the pharmacological inhibition and conditional knockout of AHR mitigated astrogliosis and microgliosis after stroke

**Fig. 3:**
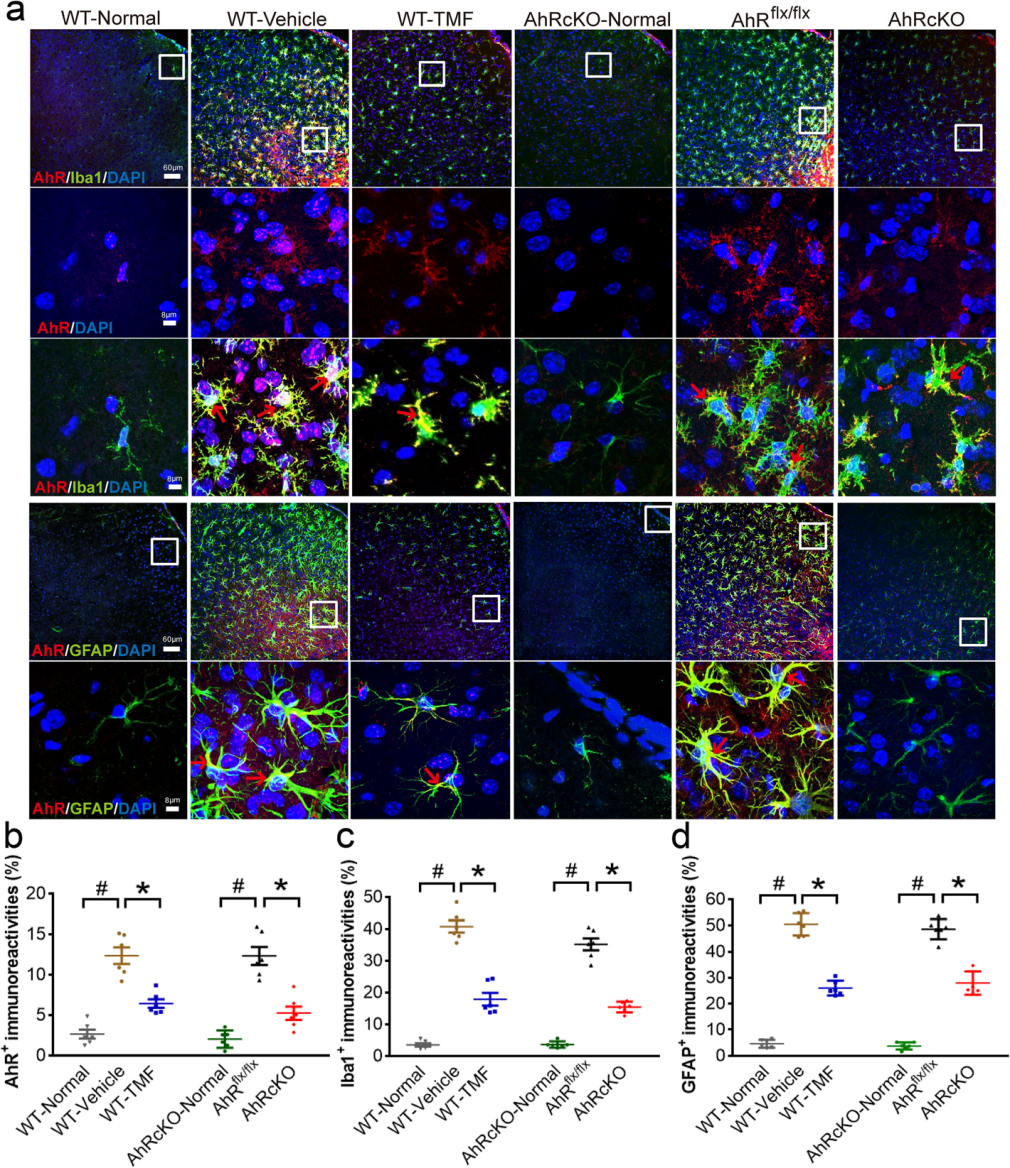
TMF-treated and AHRcKO mice showed downregulated microglia and astroglial activation at 48 h after MCAO. **a** Representative immunohistochemical staining for neuroinflammation at the peri-infarct cortex (bregma = + 0.86 mm) of normal WT (*n* = 6/each group), poststroke WT-Vehicle treated, WT-TMF treated, normal AHRcKO, poststroke AHR^flx/flx^, and AHRcKO mice. The inset in low magnification is shown with a high magnification view in the next row(s). At high magnification, note the increase in AHR immunoreactivity colocalized within Iba1-positive microglia and GFAP-positive astrocytes (red arrows) in the WT-Vehicle-treated and AHR^flx/flx^ mice after MCAO, which was markedly decreased in the WT-TMF-treated and AHRcKO mice (whose AHR spared in microglia), respectively. Furthermore, increased Iba1-positive microgliosis and GFAP-positive astrogliosis in the WT-Vehicle and AHR^flx/flx^ mice after MCAO were significantly decreased in the WT-TMF-treated and AHRcKO mice (whose AHR deleted in astroglia), respectively. Intracellular AHR immunoreactivity was predominantly in the nucleus, and to a lesser extent in the cytoplasm. Quantification of the relative immunereactive are stained with AHR (**b**), Iba-1(**c**), and GFAP (**d**). #*p* < 0.05 compared with the respective normal controls.*p < 0.05 WT-TMF compared with WT-Vehicle; AHRcKO compared with AHR^flx/flx^. The scale bars represent 60 μm at low magnification and 8 μm at high magnification

At 48 h after MCAO, immunohistochemical analyses revealed that the AHR protein was robustly expressed in ionized calcium-binding adapter molecule 1 (Iba1)-positive microgliosis and glial fibrillary acidic protein (GFAP)-positive astrogliosis predominantly at the peri-infarct cortex of the vehicle-treated WT, AHR^flx/flx^ mice compared to the normal WT and the normal AHRcKO, respectively (Fig. 3a, b). Moreover, the TMF-treated WT and AHRcKO mice showed markedly decreased GFAP-positive astrogliosis and Iba1-positive microgliosis at the peri-infarct cortex after MCAO (Fig. 3c, d). Notably, AHRcKO showed spared and reduced AHR immunoreactivities in Iba1-positive microglia (Fig. 3a) that represented the effector cell population in response to acute ischemic stroke and neuroglial deletion of AHR. These findings suggest that inhibition and the nestin^+^ cell-specific knockout of AHR reduced poststroke astrogliosis and microgliosis, indicating the reduced innate immune responses after stroke.

### Inhibition and conditional knockout of AHR increased proliferative neural progenitor cells at the ipsilesional neurogenic zones

**Fig. 4:**
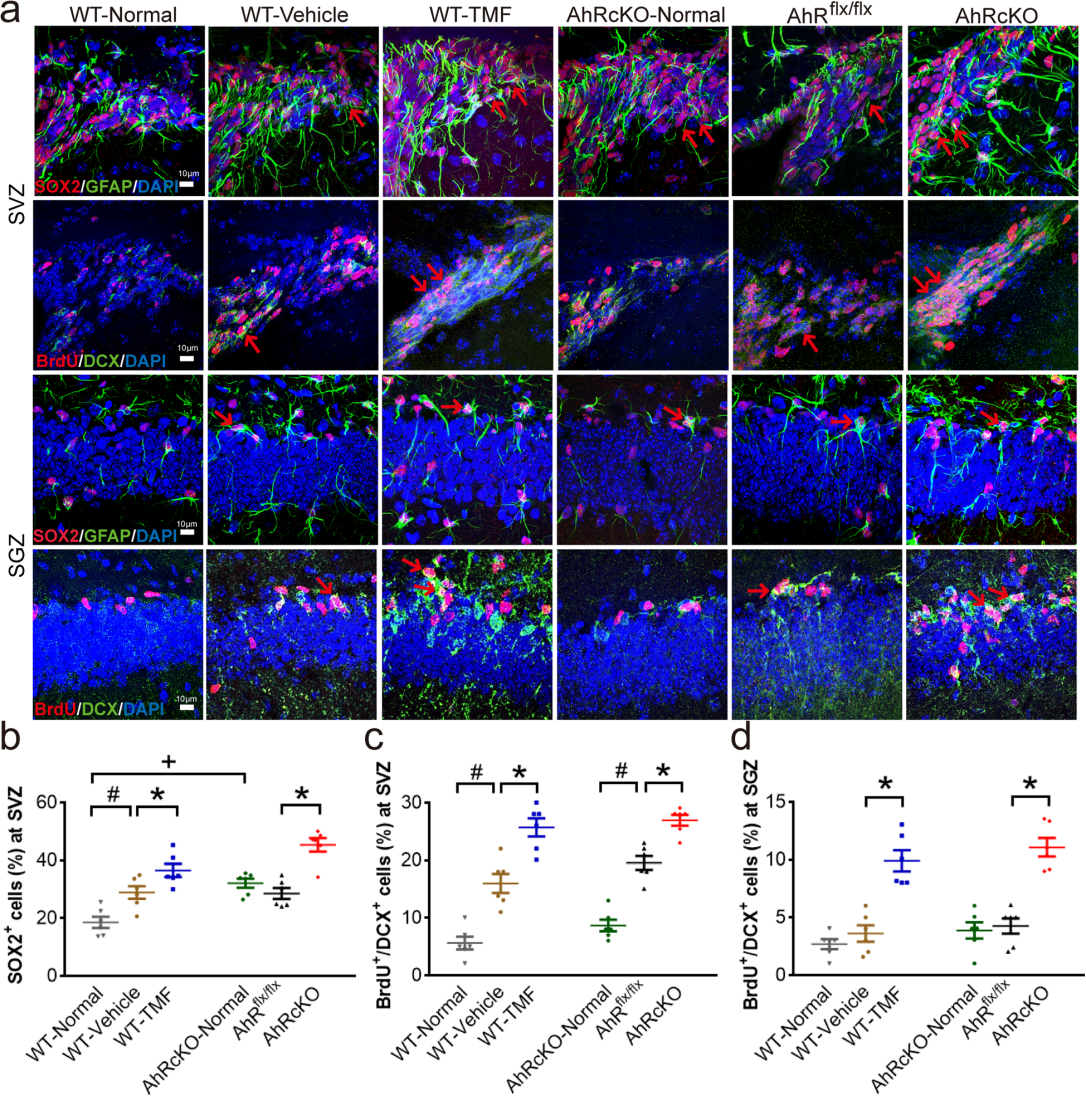
Neural stem/progenitor cells at the ipsilesional neurogenic zones at 7 days after MCAO. **a** Representative immunohistochemistry of the ipsilesional neurogenic subventricular zone (SVZ, bregma = + 0.50 mm, the upper two rows) and the hippocampal subgranular zone (SGZ, bregma = − 1.22 mm, the lower two rows) in the normal WT (*n* = 6/each group), normal AHRcKO, WT-vehicle treated, WT-TMF treated, AHR^flx/flx^, and AHRcKO mice after MCAO. **b** Quantitative immunohistochemistry of SOX2-positive cell numbers in the SVZ. Note the increased SOX2-positive cells in the SVZ of normal AHRcKO mice (red arrows). WT-TMF-treated mice and AHRcKO mice showed significantly increased SOX2-positive cells in the ipsilesional SVZ after MCAO (red arrows). **c**, **d** Quantitative immunohistochemistry of DCX- and BrdU-double-positive proliferative neural progenitor cells (NPCs) in the SVZ (**c**) and the SGZ (**d**). After MCAO, DCX- and BrdU-positive NPCs increased in the SVZ but not in the SGZ. The TMF-treated WT mice and AHRcKO mice had significantly increased newly proliferative NPCs both in the SVZ and the SGZ of the hippocampus (red arrows). #*p* < 0.05 compared with the respective normal controls. +*p* < 0.05 AHRcKO-Normal compared with WT-Normal. *p < 0.05 AHRcKO compared with AHR^flx/flx^. The scale bars represent 10 μm

To further explore whether AHR antagonist TMF treatment and nestin^+^ cell-specific AHRcKO affect neural stem cells (NSCs) and neural progenitor cells (NPCs) in adult stroke mice, we examined the number of sex determining region Y-box 2 (SOX2)-, GFAP-, and doublecortin (DCX)-expressing cells, and poststroke bromodeoxyuridine (BrdU)-labeled cells at the neurogenic subventricular zone (SVZ) and subgranular zone (SGZ) (Fig. 4a). Sox2/GFAP immunoreactivities can hardly distinguish between NSCs and poststroke reactive astrogliosis at the subventricular zone. Hence, we addressed BrdU/DCX-double positive cells for proliferative neuroblasts (NPCs). Compared with normal WT mice, the normal AHRcKO mice had more SOX2-positive cells at the SVZ. After 7 days of stroke, SOX2-positive cells and BrdU/DCX-double positive proliferative NPCs were significantly increased at the ipsilesional SVZ of the WT-Vehicle mice in line with the findings of previous studies [28, 29]. Importantly, the TMF-treated WT and AHRcKO mice had increased SOX2-positive cells compared with the respective controls at the ipsilesional SVZ (Fig. 4b), also significantly more BrdU/DCX-double positive proliferative NPCs at the ipsilesional SVZ and hippocampal SGZ compared with their respective controls (Fig. 4c, d). Thus, inhibition and nestin^+^ cell-specific AHRcKO increased proliferative NPCs at the ipsilesional neurogenic zones after MCAO, suggesting that AHR may negatively regulate poststroke neurogenesis in adult mouse brain.

### The AHR-modulated gene and protein expression related to gliosis and neurogenesis after stroke

**Fig. 5:**
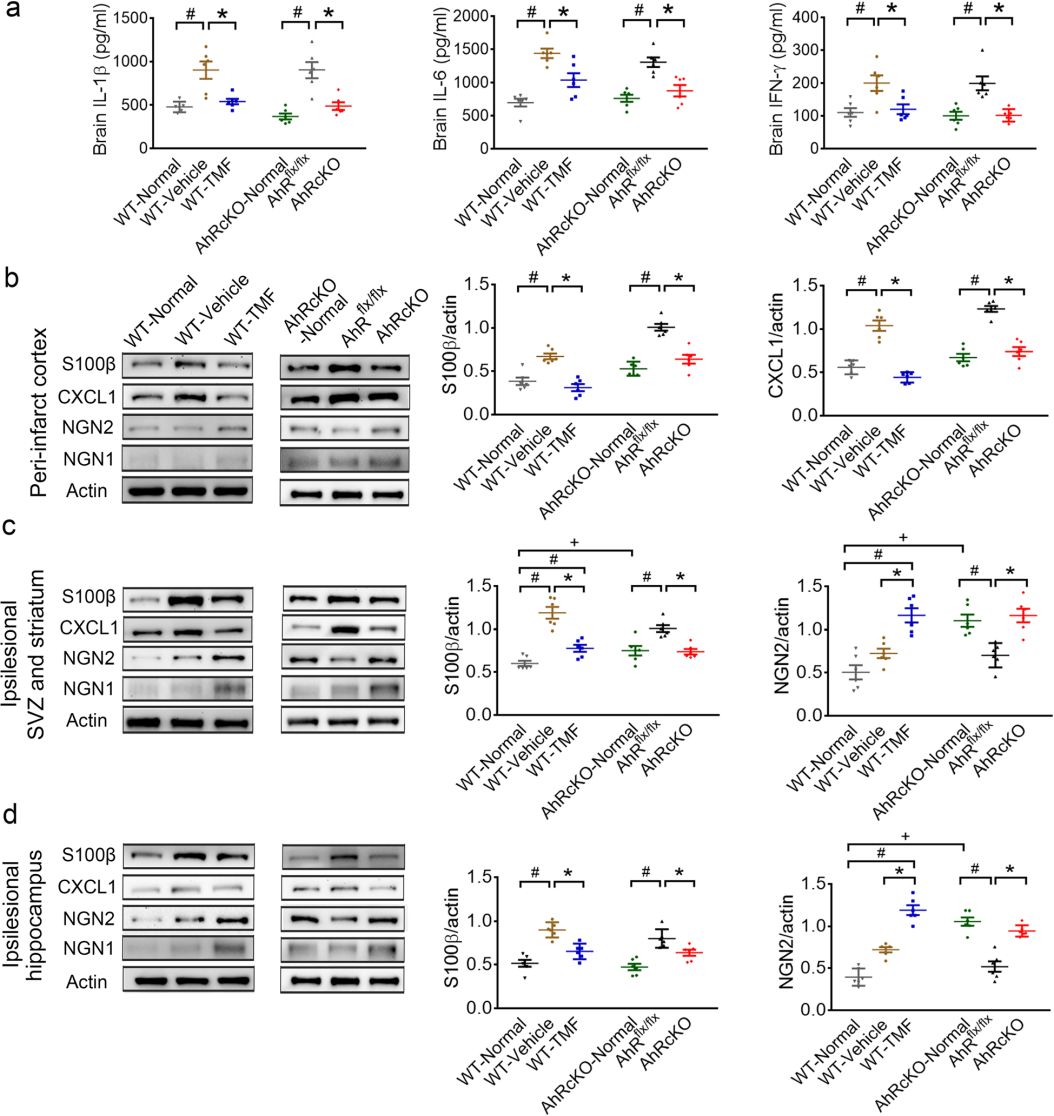
The regional cytokines and protein regulation by TMF-treatment and AHRcKO at 48 h after MCAO. **a** The levels of the proinflammatory cytokines IL-1β, IL-6, and IFN-γ from the ipsilesional hemisphere were enhanced after stroke. The WT-TMF-treated and AHRcKO mice showed attenuated IL-1β, IL-6, and IFN-γ levels in the ipsilesional hemisphere after stroke. **b**–**d** Representative western blots of the peri-infarct cortex (**b**), the ipsilesional subventricular zone (SVZ) and striatum (**c**), and the ipsilesional hippocampus (**d**) from the normal WT, poststroke WT-vehicle, WT-TMF treated mice, the normal AHRcKO, poststroke AHR^flx/flx^, and AHRcKO mice (*n* = 6/each group), with β-actin as the loading control. The WT-vehicle and AHR^flx/flx^ groups showed significantly unregulated protein levels of S100β and CXCL1 but downregulated levels in the WT-TMF group and AHRcKO mice in the peri-infarct cortex, the ipsilesional SVZ/striatum, and the ipsilesional hippocampus after MCAO. Furthermore, the protein level of NGN2 in the WT-TMF-treated and AHRcKO mice was upregulated when compared to the respective controls in the peri-infarct cortex, the ipsilesional SVZ/striatum, and the ipsilesional hippocampus after MCAO. +*p* < 0.05 AHRcKO-Normal compared with WT-Normal. #*p* < 0.05 compared with the respective normal controls. *p < 0.05 WT-TMF compared with the WT-vehicle and AHRcKO compared with the AHR^flx/flx^

The proinflammatory cytokine production of interleukin-1β (IL-1β), IL-6, and interferon-γ (IFN-γ) in the brain was upregulated in the vehicle-treated WT and AHR^flx/flx^ mice at 48 h after stroke. We found that TMF-treated WT and AHRcKO mice showed significantly attenuated IL-1β, IL-6, and IFN-γ production in the brain after stroke (Fig. 5a).

To explore the molecular mechanisms that are potentially involved in stroke-induced neurogenesis, we investigated changes in the expression of 84 array genes that are important for NSC proliferation and differentiation at the ipsilesional brain hemisphere 48 h after stroke. The comparative transcriptome analysis revealed that five gene transcripts [S100 calcium-binding protein beta (*S100β*), Chemokine (*C*-*X*-*C motif*) ligand 1 (*Cxcl1*), Bone morphogenetic protein 2 (*BMP2*), Transforming growth factor, beta 1 (*TGFβ1*), and Odd Oz/ten-m homolog 1 (*Odz1*)] were upregulated and three genes were downregulated [Myocyte enhancer factor 2C (*Mef2c*), Neurogenin 2 (*Ngn2*), and Neurogenin 1 (*Ngn1*)] after MCAO compared with the WT-Normal group (Additional file 1: Figure S2A). These eight genes were significantly counter-regulated by TMF treatment in WT stroke mice (Additional file 1: Figure S2A and S2C marked in blue). In addition, AHR^flx/flx^ mice had similarly upregulated *S100β* and *Cxcl1* expression after MCAO (Additional file 1: Figure S2B). AHRcKO mice also showed downregulated *S100β* and *Cxcl1*, and upregulated *Ngn2*, *Nuclear receptor subfamily 2*, *group E, member 3* (*Nr2e3*), and *CDK5 regulatory subunit associated protein 2* (*Cdk5rap2*) (Additional file 1: Figure S2B and S2C marked in pink)*.* Taken together, we found that *S100β*, *Cxcl1*, *Ngn2*, and *Ngn1* gene expression were commonly regulated by pharmacological inhibition and the nestin^+^ cell-specific gene deletion of AHR compared with the respective control groups (Additional file 1: Figure S2C marked in purple).

Thus, we examined the regional protein levels of these four candidates (S100β, CXCL1, NGN2, and NGN1) in the peri-infarct cortex, the ipsilesional SVZ/striatum, and the ipsilesional hippocampus at 48 h after MCAO (Fig. 5b–d). Calcium-binding protein S100β is involved in astrocytic coupling and astrogliosis, and CXCL1 is a potent neutrophil chemoattractant [28, 30]. We found that S100β and CXCL1 protein levels were elevated in the peri-infarct cortex, SVZ/striatum, and hippocampus of WT-Vehicle and AHR^flx/flx^ mice compared with their respective normal controls. Notably, these elevations were all abolished by WT-TMF treatment and AHRcKO. On the other hand, we found that NGN2, an important transcription factor for neurogenesis [29], was significantly increased in the neurogenic SVZ/striatum and the hippocampus of normal AHRcKO mice compared with normal WT mice (Fig. 5c, d). After stroke, NGN1 and NGN2 protein levels were significantly increased in the two neurogenic regions, but not the peri-infarct cortex, of the TMF-treated WT mice and AHRcKO mice compared with the AHR^flx/flx^ controls (Fig. 5b–d). Together, these findings suggest novel insights into AHR-mediated mechanisms involving astrogliosis and neurogenesis modulation. The pharmacological inhibition and neuroglial cell-specific deletion of AHR improved functional outcomes of acute ischemic stroke, which were more likely contributed by neuroprotection, ameliorated astrogliosis, and microglial infiltration, rather than increased neurogenesis at the ipsilesional neurogenic SVZ and the hippocampus.

## Discussion

In this study, we reported that nestin^+^ cell-specific AHR conditional knockout (AHRcKO) mice, whose AHR are deleted from neural stem/progenitor cells and also in all their lineage, exhibit normal physical and behavioral phenotypes (Fig. 1c) in contrast to AHR-null mice that suffer from multiple system pathologies [12, 22, 31]. Therefore, nestin^+^ cell-specific AHRcKO mice are ideal for investigating the roles of AHR signaling in physiological and pathological functions in the brain. Here, we demonstrate that the nestin^+^-cell-specific AHRcKO and the pharmacological inhibition of AHR protect sensorimotor and memory deficits against acute cerebral ischemia and exert regulatory effects on anti-inflammation and adult neurogenesis. AHR activation points to damaging roles of acute ischemic inflammation and gliosis in stroke pathophysiology. Further studies are required to elucidate the molecular mechanisms of AHR-regulated astrogliosis and neurogenesis. Our findings are in line with the previous report that the kynurenine/AHR activation mediated acute ischemic injury using the AHR-null mice and the cortical neuronal cultures subjected to oxygen-glucose deprivation [13]. Moreover, nestin is expressed not only in neural stem/progenitor cells but also in developing, not mature, endothelial cells and cardiac myocytes [32–34]. We did not record the blood pressure in our TMF-treated mice or constitutive Nestin-Cre AHRcKO. The systemic effect of AHR inhibition on blood pressure needs further investigation [35].

The kynurenine/AHR pathway is tightly controlled by the immune system. Both systemic and brain inflammatory conditions stimulate this pathway, including cytokine-induced IDO activation, thus contributing to kynurenine/AHR activation in several neurological disorders [36]. AHR has been shown to exhibit either proinflammatory or anti-inflammatory activity in a context-specific manner [16, 37]. The switch from the proinflammatory to the anti-inflammatory mode in response to different ligands allows AHR to play different roles in the regulation of immune responses [16, 17]. In the experimental autoimmune encephalomyelitis model, CX3CR1^+^ microglia-specific AHR deletion upregulated the pro-inflammatory astrocytes [23], and GFAP^+^ astrocyte-specific AHR deletion also increased inflammatory cytokines expression [37, 38], suggesting that both astrocytic and microglia AHR may limit chronic autoimmune inflammation. In our stroke model, by contrast, nestin^+^ cell-specific AHRcKO mice and TMF treatment inhibited AHR and reduced acute ischemic inflammation. In our AHRcKO mice, microglia were spared from AHR deletion, therefore neuroglial AHR may play a primary role in acute ischemic inflammation. AHR activation affects the expression of multiple immunoregulatory genes and the function of different inflammatory cells [39]. Several inflammatory response-related genes contain multiple DREs in their upstream sequences [40]. For example, AHR occupies the enhancer region of the IL-6 promoter, suggesting a role for AHR in IL-6 signaling [41]. Further experiments and other cell type-specific deletion of AHR are needed in stroke settings to elucidate the AHR-dependent mechanisms.

Inflammation-associated conditions have been reported to impair neurogenesis, neuronal survival, and differentiation in the mammalian brain [42]. The proinflammatory functions of IL-1β and IFN-γ decreased hippocampal NSC proliferation and increased neuronal apoptosis [43, 44]. Therefore, the attenuated inflammation by AHR inhibition may indirectly support neurogenesis after stroke. Interestingly, AHR was recently identified as a crucial regulator of restorative neurogenesis in the zebrafish brain, which has a high capacity to replace brain neurons generated by a specific set of ependymoglial cells after injury [45]. Low AHR signaling triggered restorative ependymoglial proliferation after acute injury, while high AHR signaling promoted ependymoglial differentiation toward post-mitotic neurons after injury [45]. Also, another study reported that tamoxifen-inducible Nestin-Cre AHR deletion after postnatal day 30 increased adult hippocampal neurogenesis in nonstroke mice [46], which is in line with our findings that AHR plays a role in regulation of adult neurogenesis. However, the function of adult-born neurons after stroke remains unknown. The AHR-mediated neurogenic effect was less likely to contribute to the enhanced working memory functions by 7 days after stroke compared to the anti-inflammatory effects. Further investigation on the pathophysiological mechanisms of poststroke neurogenesis and functional integration into neural networks should be addressed.

## Conclusions

We demonstrated the AHR signaling and its actions on astrogliosis and neurogenesis in acute ischemic stroke, which were not described previously. Both the pharmacological inhibition of AHR by TMF and the nestin^+^ cell-specific knockout of AHR significantly ameliorated ischemic brain infarction, sensorimotor deficits, and nonspatial working memory, in association with a significant reduction in astrogliosis and microglial infiltration. Interestingly, we disclosed enhanced endogenous neurogenesis at the ipsilesional neurogenic zones, i.e., the SVZ and the hippocampal SGZ, in TMF-treated WT and AHRcKO mice. Moreover, AHR inhibition and AHRcKO both affected proinflammatory cytokines IL-1β, IL-6, IFN-γ, CXCL1, as well as S100β, NGN2, and NGN1 gene and protein expression. Together, these findings showed that AHR signaling potentially mediates poststroke gliosis and ischemic brain injuries. Timely inhibition of AHR signaling may benefit not only anti-inflammatory but also neurogenic effects in acute ischemic stroke.

## Additional file

Additional file 1: Figure S1 Representative immunohistochemical staining for AHR intracellular distribution. Depending on the status of AHR expression and the availability of AHR ligands, which is presumably different among cells, AHR ligands would bind to cytoplasmic AHR for its activation and nuclear translocation dynamically. Compared with the normal AHRcKO which had little AHR immunoreactivity (B), the normal AHRflx/flx (A) showed predominantly nuclear distribution of AHR immunoreactivity, and to a lesser extent in the cytoplasm in Iba1-positive and Iba-1 negative cells. Figure S2 The expression levels of 84 candidate genes and 5 housekeeping genes from the ipsilesional hemisphere by neurogenesis array. (A) The array data obtained by real-time polymerase chain reaction (RT-PCR). Changes beyond 1 ± 0.5-fold are shown as differentially expressed genes. The WT-Vehicle group show the upregulated gene expression of S100β, Cxcl1, Bmp2, Tgfβ1, Odz1 and downregulated Mef2c, Ngn2 and Ngn1 at 48 h after MCAO. In contrast, the WT-TMF group downregulated the gene expression of S100β, Cxcl1, Bmp2, Tgfβ1, and Odz1 and upregulated Mef2c, Ngn2 and Ngn1 compared with vehicle treatment after MCAO. (B) On the other hand, in the AhRflx/flx group, upregulated S100β and Cxcl1 gene expression was observed after MCAO. In AhRcKO mice, downregulated S100β and Cxcl1 and upregulated Ngn2, Nr2e3 and Cdk5rap2 gene expression were noted compared with the AhRflx/flx group after MCAO (n = 3/each group). (C) In summary of the 84 gene expression regulation, the common changes by pharmacological inhibition (TMF, marked in blue) and AhRcKO mice (marked in pink) were S100β, Cxcl1, Ngn2, and Ngn1 (marked in purple). #*p* < 0.05 compared with the respective normals. *p < 0.05 WT-TMF compared with the WT-Vehicle and AhRcKO compared with the AhRflx/flx. (DOCX 1757 kb)

## Data Availability

The data that support the findings are not publicly available. Data are available from the authors upon reasonable request.

## Abbreviations

AHR: Aryl hydrocarbon receptor
AHRcKO: AHR conditional knockout
ARNT: AHR nuclear translocator
BDNF: Brain-derived neurotrophic factor
BMP2: Bone morphogenetic protein 2
Cdk5rap2: CDK5 regulatory subunit associated protein 2
COX-2: Cyclooxygenase-2
CXCL1: Chemokine (C-X-C motif) ligand 1
DCX: Doublecortin
DRE: Dioxin response elements
FICZ: 6-Formylindolo (3,2-b)carbazole
GFAP: Glial fibrillary acidic protein
Iba1: Ionized calcium-binding adapter molecule 1
IDO: Indoleamine 2,3-dioxygenase
IFN-γ: Interferon gamma
IL-1β: Interleukin 1 beta
IL-6: Interleukin 6
iNOS: Inducible nitric oxide synthase
MCAO: Middle cerebral artery occlusion
Mef2c: Myocyte enhancer factor 2C
Ngn1: Neurogenin 1
Ngn2: Neurogenin 2
NPC: Neural progenitor cell
Nr2e3: Nuclear receptor subfamily 2, group E, member 3
NSC: Neural stem cell
Odz1: Odd Oz/ten-m homolog 1
PBS: Phosphate-buffered saline
PBST: Phosphate-buffered saline
RT-PCR: Real-time polymerase chain reaction
S100β: S100 calcium-binding protein beta
SGZ: Subgranular zone
SOX2: Sex determining region Y-box 2
SVZ: Subventricular zone
TDO: Indoleamine 2,3-dioxygenase
TGFβ1: Transforming growth factor, beta 1
TMF: 6,2′,4′-trimethoxyflavone
TTC: 2,3,5-triphenyltetrazolium chloride
WT: Wild-type
