# Supplementary material for: Aryl hydrocarbon receptor modulates stroke-induced astrogliosis and neurogenesis in the adult mouse brain
Source: J Neuroinflammation. 2019 Oct 12;16:187. doi: 10.1186/s12974-019-1572-7 (PMC6790016; doi:10.1186/s12974-019-1572-7)
Supplement: Supplementary file 1 — Figure S1 Representative immunohistochemical staining for AHR intracellular distribution. Depending on the status of AHR expression and the availability of AHR ligands, which is presumably different among cells, AHR ligands would bind to cytoplasmic AHR for its activation and nuclear translocation dynamically. Compared with the normal AHRcKO which had little AHR immunoreactivity (B), the normal AHRflx/flx (A) showed predominantly nuclear distribution of AHR immunoreactivity, and to a lesser extent in the cytoplasm in Iba1-positive and Iba-1 negative cells. Figure S2 The expression levels of 84 candidate genes and 5 housekeeping genes from the ipsilesional hemisphere by neurogenesis array. (A) The array data obtained by real-time polymerase chain reaction (RT-PCR). Changes beyond 1 ± 0.5-fold are shown as differentially expressed genes. The WT-Vehicle group show the upregulated gene expression of S100β, Cxcl1, Bmp2, Tgfβ1, Odz1 and downregulated Mef2c, Ngn2 and Ngn1 at 48 h after MCAO. In contrast, the WT-TMF group downregulated the gene expression of S100β, Cxcl1, Bmp2, Tgfβ1, and Odz1 and upregulated Mef2c, Ngn2 and Ngn1 compared with vehicle treatment after MCAO. (B) On the other hand, in the AhRflx/flx group, upregulated S100β and Cxcl1 gene expression was observed after MCAO. In AhRcKO mice, downregulated S100β and Cxcl1 and upregulated Ngn2, Nr2e3 and Cdk5rap2 gene expression were noted compared with the AhRflx/flx group after MCAO (n = 3/each group). (C) In summary of the 84 gene expression regulation, the common changes by pharmacological inhibition (TMF, marked in blue) and AhRcKO mice (marked in pink) were S100β, Cxcl1, Ngn2, and Ngn1 (marked in purple). #p < 0.05 compared with the respective normals. *p < 0.05 WT-TMF compared with the WT-Vehicle and AhRcKO compared with the AhRflx/flx. (DOCX 1757 kb) [file 12974_2019_1572_MOESM1_ESM.docx]

**ADDITIONAL FILE 1**

**Aryl Hydrocarbon Receptor Modulates Stroke-induced Astrogliosis and Neurogenesis in the Adult Mouse Brain**

Wan-Ci Chen^1^, MSc; Li-Hsin Chang^2^, BS; Shiang-Suo Huang^3^, PhD; Yu-Jie Huang^1^, BS; Chun-Lien Chih^4^, AS; Hung-Chih Kuo^5^, PhD; Yi-Hsuan Lee^1*^, PhD; I-Hui Lee^2, 6*^, MD, PhD

^1^Department and Institute of Physiology, National Yang-Ming University, Taipei, Taiwan; ^2^Institute of Brain Science, Brain Research Center, National Yang-Ming University, Taipei, Taiwan; ^3^Department of Pharmacology, Institute of Medicine, Chung-Shan Medical University, Taichung, Taiwan; ^4^Cheng-Hsin General Hospital, Taipei, Taiwan; ^5^Stem Cell Program, Institute of Cellular and Organismic Biology, Academia Sinica, Taipei, Taiwan; ^6^Division of Cerebrovascular Diseases, Neurological Institute, Taipei Veterans General Hospital, Taipei, Taiwan

***Contributed equally**

**Corresponding author**:

I-Hui Lee, MD, PhD

Address: No.201, Sec. 2, Shipai Rd., Beitou District, Taipei City, Taiwan 11217

Phone: +886-2-28712121 ext.8109

Fax: +886-2-28757579

E-mail address: [ihlee@vghtpe.gov.tw](mailto:ihlee@vghtpe.gov.tw)

Yi-Hsuan Lee, PhD

Address: No.155, Sec. 2, Linong Street, Beitou District, Taipei City, Taiwan 11217

Phone: +886-2-28267067

Fax: +886-2-28264049

E-mail address: [yhlee3@ym.edu.tw](mailto:yhlee3@ym.edu.tw)


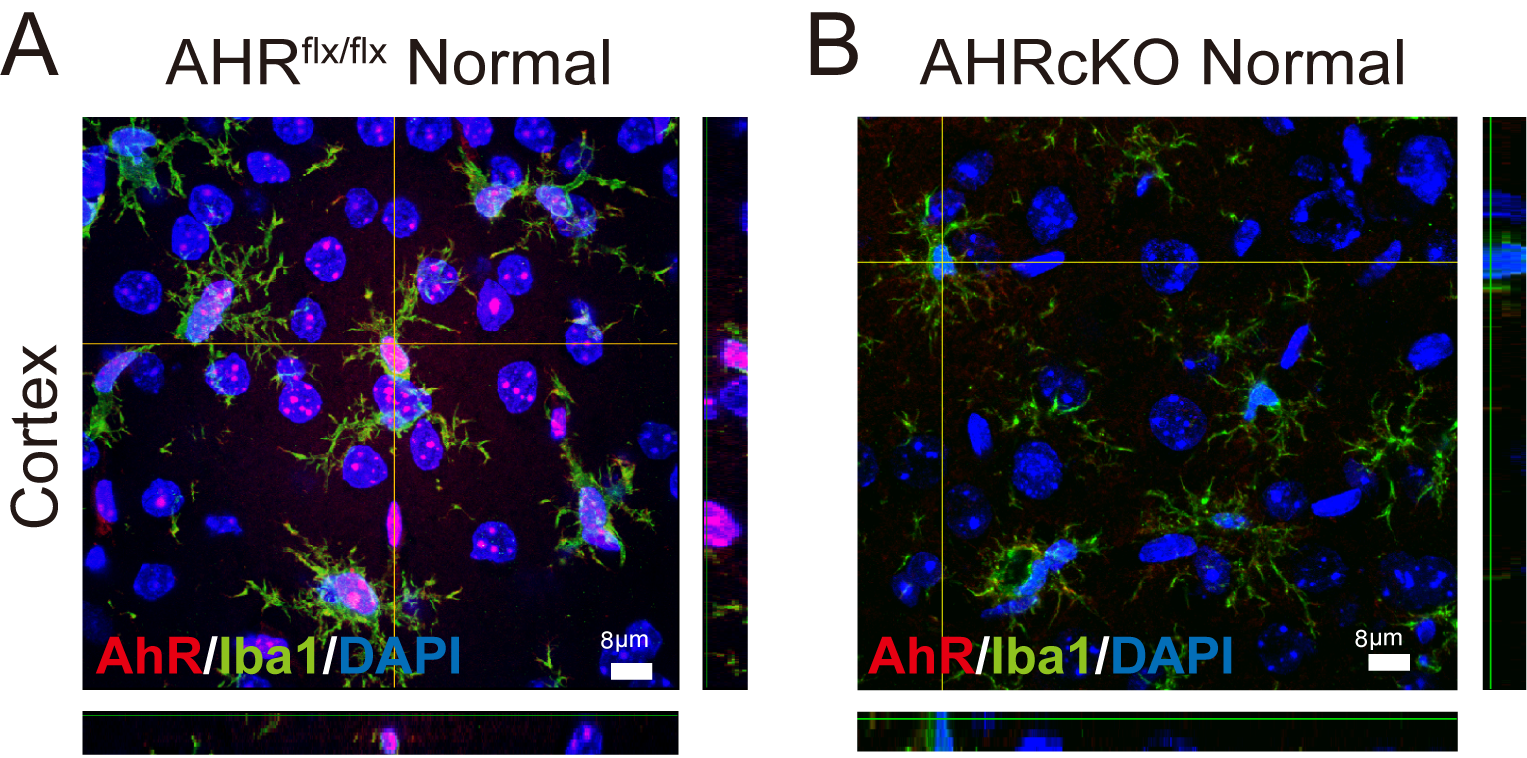


**Figure S1. Representative immunohistochemical staining for AHR intracellular distribution.**

Depending on the status of AHR expression and the availability of AHR ligands, which is presumably different among cells, AHR ligands would bind to cytoplasmic AHR for its activation and nuclear translocation dynamically. Compared with the normal AHRcKO which had little AHR immunoreactivity (B), the normal AHR^flx/flx^ (A) showed predominantly nuclear distribution of AHR immunoreactivity, and to a lesser extent in the cytoplasm in Iba1-positive and Iba-1 negative cells.

**
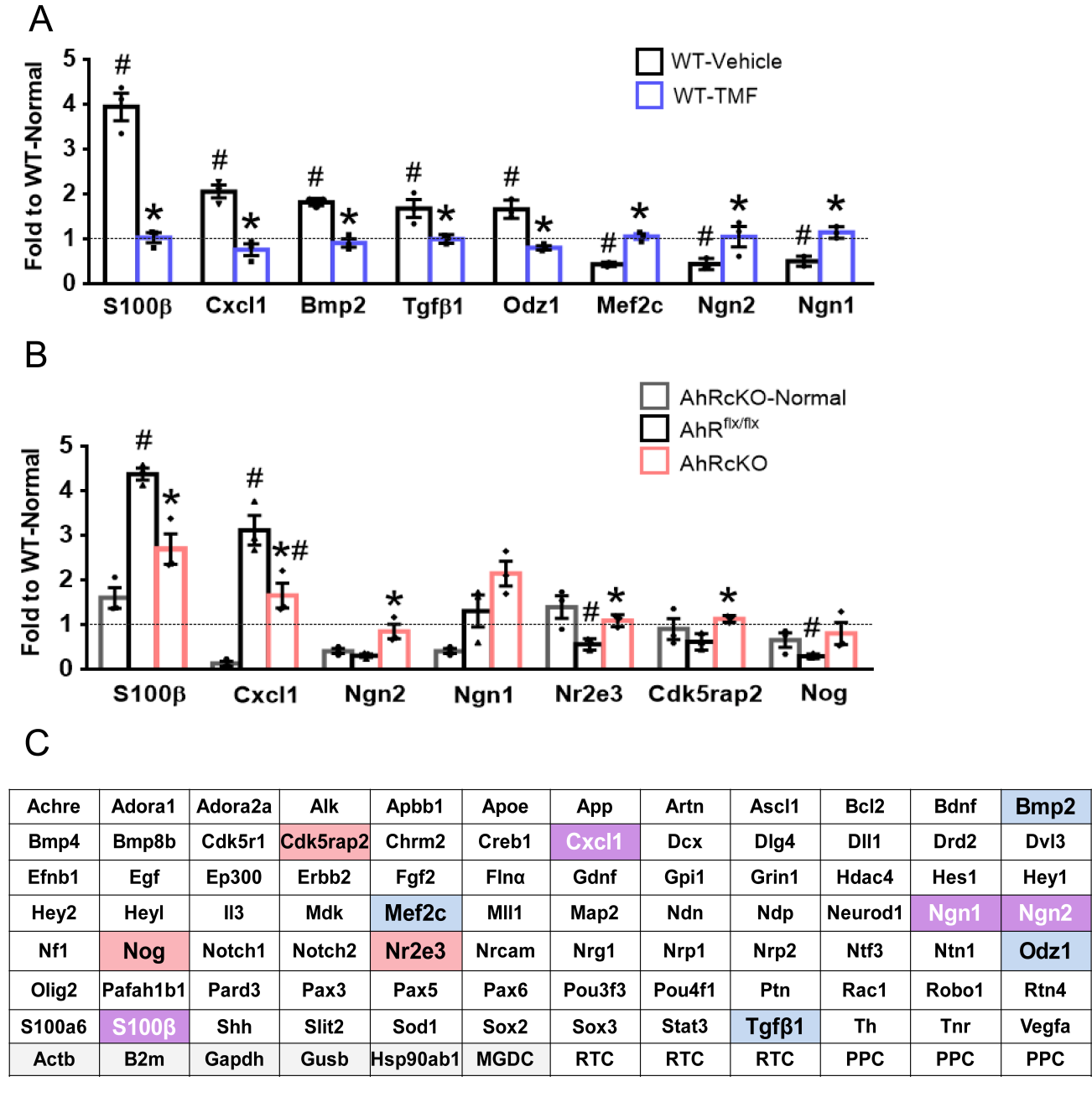
**

**Figure S2. The expression levels of 84 candidate genes and 5 housekeeping genes from the ipsilesional hemisphere by neurogenesis array.**

(A) The array data obtained by real-time polymerase chain reaction (RT-PCR). Changes beyond 1[±](https://tw.answers.yahoo.com/question/index?qid=20070702000016KK08383&p=%E6%AD%A3%E8%B2%A0)0.5-fold are shown as differentially expressed genes. The WT-Vehicle group show the upregulated gene expression of *S100β*, *Cxcl1*, *Bmp2*, *Tgfβ1*, *Odz1* and downregulated *Mef2c*, *Ngn2* and *Ngn1* at 48 hours after MCAO. In contrast, the WT-TMF group downregulated the gene expression of *S100β*, *Cxcl1*, *Bmp2*, *Tgfβ1*, and *Odz1* and upregulated *Mef2c*, *Ngn2* and *Ngn1* compared with vehicle treatment after MCAO. (B) On the other hand, in the AhR^flx/flx^ group, upregulated *S100β* and *Cxcl1* gene expression was observed after MCAO. In AhRcKO mice, downregulated *S100β and Cxcl1* and upregulated *Ngn2, Nr2e3* and *Cdk5rap2* gene expression were noted compared with the AhR^flx/flx^ group after MCAO (n=3/each group). (C) In summary of the 84 gene expression regulation, the common changes by pharmacological inhibition (TMF, marked in blue) and AhRcKO mice (marked in pink) were *S100β*, *Cxcl1*, *Ngn2,* and *Ngn1* (marked in purple). #p<0.05 compared with the respective normals. *p<0.05 WT-TMF compared with the WT-Vehicle and AhRcKO compared with the AhR^flx/flx^.
